# Open-loop amplitude-modulation Kelvin probe force microscopy operated in single-pass PeakForce tapping mode

**DOI:** 10.3762/bjnano.12.83

**Published:** 2021-10-06

**Authors:** Gheorghe Stan, Pradeep Namboodiri

**Affiliations:** 1 Material Measurement Laboratory, National Institute of Standards and Technology, Gaithersburg, MD 20899, USA; 2 Physical Measurement Laboratory, National Institute of Standards and Technology, Gaithersburg, MD 20899, USA

**Keywords:** electrostatic interaction, Kelvin probe force microscopy, open loop, surface potential

## Abstract

The open-loop (OL) variant of Kelvin probe force microscopy (KPFM) provides access to the voltage response of the electrostatic interaction between a conductive atomic force microscopy (AFM) probe and the investigated sample. The measured response can be analyzed a posteriori, modeled, and interpreted to include various contributions from the probe geometry and imaged features of the sample. In contrast to this, the currently implemented closed-loop (CL) variants of KPFM, either amplitude-modulation (AM) or frequency-modulation (FM), solely report on their final product in terms of the tip–sample contact potential difference. In ambient atmosphere, both CL AM-KPFM and CL FM-KPFM work at their best during the lift part of a two-pass scanning mode to avoid the direct contact with the surface of the sample. In this work, a new OL AM-KPFM mode was implemented in the single-pass scan of the PeakForce Tapping (PFT) mode. The topographical and electrical components were combined in a single pass by applying the electrical modulation only in between the PFT tip–sample contacts, when the AFM probe separates from the sample. In this way, any contact and tunneling discharges are avoided and, yet, the location of the measured electrical tip–sample interaction is directly affixed to the topography rendered by the mechanical PFT modulation at each tap. Furthermore, because the detailed response of the cantilever to the bias stimulation was recorded, it was possible to analyze and separate an average contribution of the cantilever to the determined local contact potential difference between the AFM probe and the imaged sample. The removal of this unwanted contribution greatly improved the accuracy of the AM-KPFM measurements to the level of the FM-KPFM counterpart.

## Introduction

Over many years, an abundance of developments and applications has made Kelvin probe force microscopy (KPFM) [1] one of the most versatile nanoscale surface electronic characterization techniques. With its main measurement in terms of the local contact potential difference (CPD) between a conductive AFM probe and a surface, KPFM has been used for qualitative and quantitative electric characterizations. Examples include surface potential, doping, charge profiling, optoelectronic response, and others on various materials and structures including metals [1], semiconductors [2–4], dielectrics [5–7], photovoltaics [8–10], polymers [11–13], ferroelectrics [14–16], and biological samples [17–19]. Technical descriptions and applications of KPFM methods for nanoscale material property characterizations are found in many review articles and book chapters [13,20–24].

The majority of the KPFM implementations are in the form of closed-loop systems, with the tip–sample CPD determined from the nullification [25] of either the electrostatic force as in AM-KPFM [1,26] or the gradient force as in FM-KPFM [27–28]. The benefit of a CL KPFM method is that the CPD is readily obtained in the form of a final product that is assembled in a map over the scanned area. However, the detailed response of the electrostatic tip–sample interaction is not available in CL KPFM, so post-processing and modeling of data is limited. Moreover, the finite response time of the CL feedback (of the order of milliseconds in some cases) prevents the use of CL KPFM from observing fast electrodynamic processes. Some of these impediments are addressed in OL implementations such as time-resolved electrostatic force microscopy [29–30], pump–probe KPFM [31–32], or fast free force recovery KPFM [33] that are capable of observing the dynamics of the optoelectronic response of materials and electric field-induced charge migration at time scales of the order of tens of microseconds.

Various OL KPFM implementations with operation on either an AM or FM modulation have been demonstrated [34–36]. They incorporate a direct measurement of either the amplitude or frequency response of the AFM probe to an applied single-frequency bias modulation. Furthermore, multi-frequency operations of OL KPFM were introduced as band-excitation OL BE-KPFM [37–39], intermodulation electrostatic force microscopy [40], and dual-harmonic KPFM (DH-KPFM) [34,41–42]. In DH-KPFM, the CPD is obtained from the ratio of the amplitudes of the first two harmonics of the cantilever response to an AC bias modulation and requires a prior calibration for the gain of the cantilever’s transfer function. DH-KPFM has found applications on sensitive materials and solid–liquid interfaces where conventional CL KPFM does not perform very well [43]. CPD measurements in an OL operation have also been demonstrated in more inclusive scanning probe modes such as the general acquisition (G-mode) KPFM [35,44–46] with sampling rates of the order of megahertz. The high-speed data acquisition brings a substantial increase in the spatial and temporal resolutions of the measurements. Thus, a reported CPD measurement of the G-mode KPFM was on a time scale of the order of 20 μs, which is about one hundred times faster than CL KPFM modes and could be used to observe ultrafast dynamics of electrical processes [35,46].

Due to the long-range nature of the electrostatic interactions, contributions from all the conductive parts (apex, cone, and cantilever) of an AFM probe accumulate to influence the CPD measured by KPFM. In AM-KPFM implementations especially, the CPD values are receptive to non-local capacitive couplings that degrade the tip-confinement sensitivity over heterogeneous samples [25,36,47]. Conversely, FM-based KPFM methods operate on the gradient of the tip–sample electrostatic force and are less sensitive to the stray capacitive couplings outside the immediate vicinity of the AFM tip [48]. This increases the measurement accuracy of FM-KPFM in both CL and OL configurations [36,49–50]. It has also been demonstrated that the spatial resolution and measurement sensitivity of the AM-KPFM can be increased by deconvoluting the capacitive couplings. Corrections to the measured CPD values are based on multi-capacitances description [51], point spread function deconvolution [47,52–53], electrodynamic models [54], and numerical modeling [55] of the tip–sample system. While in CL AM-KPFM these deconvolutions apply to the CPD maps as inverse problems, it is conceivable that a simple model such as a multi-capacitances approximation can be run on OL AM-KPFM measurements to remove some of the stray capacitive couplings concomitantly with the CPD extraction.

In this work, a new OL AM-KPFM method was used to acquire the full response of an AFM probe during a single-pass intermittent contact mode. The bias modulation was synchronized with the oscillations of the PeakForce Tapping (PFT) mode [56] and selectively applied only during the out-of-contact intervals of the PFT motion. Because this method consists of a single-pass scan, the CPD determined from the acquired data at a given location can be directly affixed to the topography provided by PFT at that location; in two-pass KPFM scans, the CPD trace determined in the second pass is distributed over the topography line recorded in the first pass. Also, because the response to the applied bias modulation was fully acquired in the proposed OL KPFM implementation, the CPD was determined by modeling the electrostatic interaction between the AFM probe and the sample. This was done either on the parabolic bias dependence of the AFM deflection [33] or by analyzing the time series response of the AFM deflection to the applied bias [36]. In both analyses, the average contribution of the capacitive coupling of the AFM cantilever to the electrostatic interaction was separated by calibrating the local CPD on sample regions of known surface potential. This simple and practical deconvolution increased the spatial resolution of the OL AM-KPFM at the level of an FM-KPFM method. The OL KPFM variant proposed here adds to a growing set of PFT-based platform techniques that includes electrical [57–58], chemical [59], optical [60–61], and mechanical [62–63] measurements.

## Results and Discussion

### Closed-loop KPFM measurements in two-pass PFT mode

**Figure 1:**
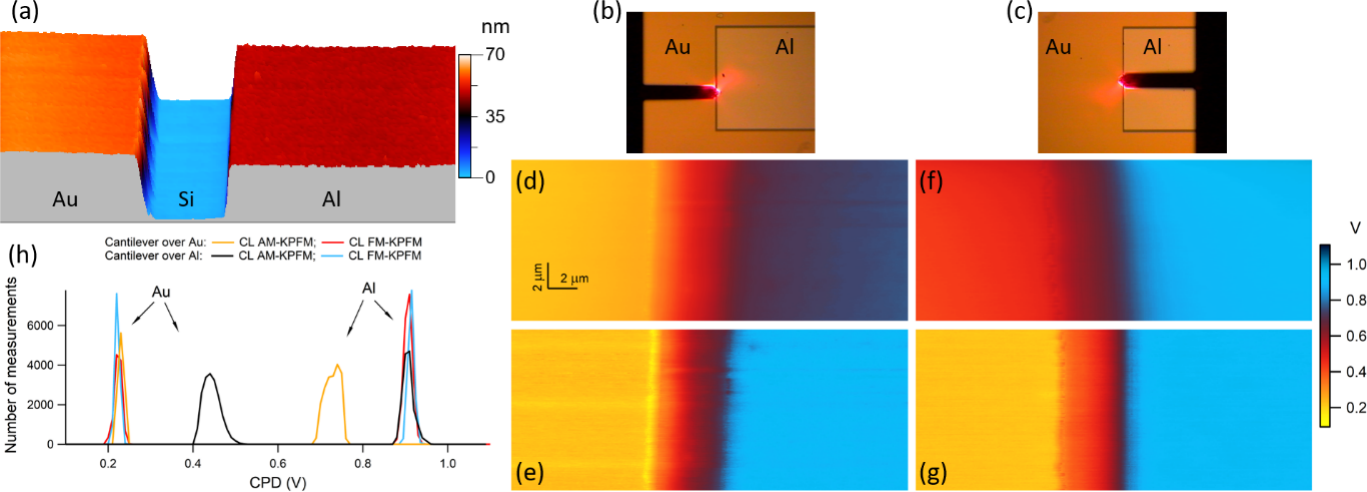
CL KPFM measurements over an Au/Si/Al trench: (a) topography; (b) optical view of the AFM probe during a scan with the cantilever over the Au region; (c) optical view of the AFM probe during a scan with the cantilever over the Al region; (d, e) CPD maps from CL AM-KPFM and CL FM-KPFM scans, respectively, in the configuration shown in (b); (f, g) CPD maps from CL AM-KPFM and CL FM-KPFM scans, respectively, in the configuration shown in (c); (h) histograms of the measured CPD over the Au and Al regions of the maps shown in (d–g). The maps in (d–g) share the same color scale shown on the right of the figure.

The new OL AM-KPFM implementation was tested on a commercially available sample consisting of large Au and Al metal regions deposited on a Si substrate (Bruker Nano Surfaces, Santa Barbara, CA, USA); the metal regions are separated by trenches that expose the Si substrate at their bottom. Figure 1a shows the AFM topographical image of one of these trenches, bordered by Au (left) and Al (right). The CPD maps over the sample were obtained first by using two common CL KPFM modes that are implemented on PFT, namely CL AM-KPFM and CL FM-KPFM [57]. Both these CL modes are two-pass scanning modes with topography acquired in the first pass by PFT and the KPFM measurements performed in the second pass at a constant height above the surface (lift height), with the AFM probe following the line topography acquired in the first pass. All the KPFM discussed in this work were made with the sample grounded and the electrical modulation routed through the conductive tip. The AFM probe, same for both CL-KPFM and OL-KPFM measurements, was a SCM-PIT V2 PtIr coated probe (Bruker, Santa Barbara, CA, USA).

A set of CPD maps over the same Au/Si/Al trench is shown in Figure 1d–g. As can be seen, the CPD contrast of the maps obtained by CL AM-KPFM changes with the position of the cantilever over either Au or Al (refer to Figure 1d–f) whereas the CPD maps from CL FM-KPFM are insensitive to the selected tip–sample orientation. Thus, when the cantilever is over the Au region (Figure 1b), the CPD measured over Au is the same in both AM-KPFM (left part of Figure 1d) and FM-KPFM (left part of Figure 1e) but significantly lower over the Al region in the AM-KPFM map (right part of Figure 1d) than in its counterpart of the FM-KPFM map (right part of Figure 1e). The situation reverses when the cantilever overlooks the Al region (Figure 1c). In this case, the CPD measured by AM-KPFM over the Al (right part of Figure 1f) is the same as that measured by FM-KPFM (right part of Figure 1g) and is higher over the Au part (left parts of Figure 1f,g). The histograms of the CPD values over the two metallic regions are summarized in Figure 1h and show that the absolute value of the CPD between the Au and Al regions is about 0.7 eV from FM-KPFM and about 0.5 eV from AM-KPFM measurements.

The observed difference in the CPD measured by these two CL KPFM modes is well documented [36,50–51,58] and is related to the physical quantity on which each mode operates. On one hand, the feedback loop of the CL AM-KPFM tries to nullify the magnitude of the electrostatic force developed between the AFM probe and sample. Because the entire AFM probe (tip and cantilever) is conductive, a significant contribution to this force comes from the capacitive coupling between the cantilever and the sample region underneath the cantilever. As such, when the tip images a region with a surface potential significantly different than that of the region above which the cantilever floats (e.g., tip over the Au region and cantilever over the Al region), the CPD measured by AM-KPFM has a strong delocalized component from the cantilever–sample interaction. On the other hand, the cantilever–sample capacitive coupling is highly mitigated in CL FM-KPFM variants, which operate on nullifying the electrostatic force gradient between tip and sample. In KPFM gradient detection methods, the almost linear distance-dependent force between cantilever and sample is mostly removed from the measured CPD [47,50,64].

The above discussion suggests that the measurement accuracy of AM-KPFM could be greatly improved once the contribution of the cantilever is accounted for and removed from the measured CPD. A separation of the various contributions to the measured CPD however would require a data analysis after the measurements are made and most likely access to the raw response of the probe under an electrostatic interaction with the sample. These requirements are very hard to be fulfilled by a CL KPFM method, where the momentarily reported CPD is the result of a feedback loop algorithm. However, the data are fully observed and recorded in OL KPFM measurements and a posteriori analysis can be more inclusive and customized to a given measurement setup.

### Open-loop AM-KPFM measurements and data analysis in one-pass PFT mode

Various OL KPFM methods have been introduced as viable alternatives to the existing CL KPFM methods. One of the main motivations for OL KPFM is the full access to the raw data, uncorrected by any measurement procedure such as the feedback loop of a real-time operation. Moreover, the limited speed response of the feedback loop can also become a great impediment in observing ultrafast phenomena at time scale of the order of microseconds or less [29–33].

The most direct and easy implementations of OL-KPFM are in terms of amplitude modulation due to the intrinsic force detection that the AFM operates on. The OL-KPFM developed in this work is an AM method that performs CPD measurements in tandem with the progressive tracing of the topography at each location. This was accomplished by applying short sinusoidal bias signals during the out-of-contact time intervals of the PFT modulation. Thus, rather than the more common two-pass KPFM measurements, this new implementation is carried out in one-pass, with the CPD value affixed to the topographical feature and other PFT quantities (e.g., elastic modulus, pull-off force, and dissipation) at each location during scanning.

**Figure 2:**
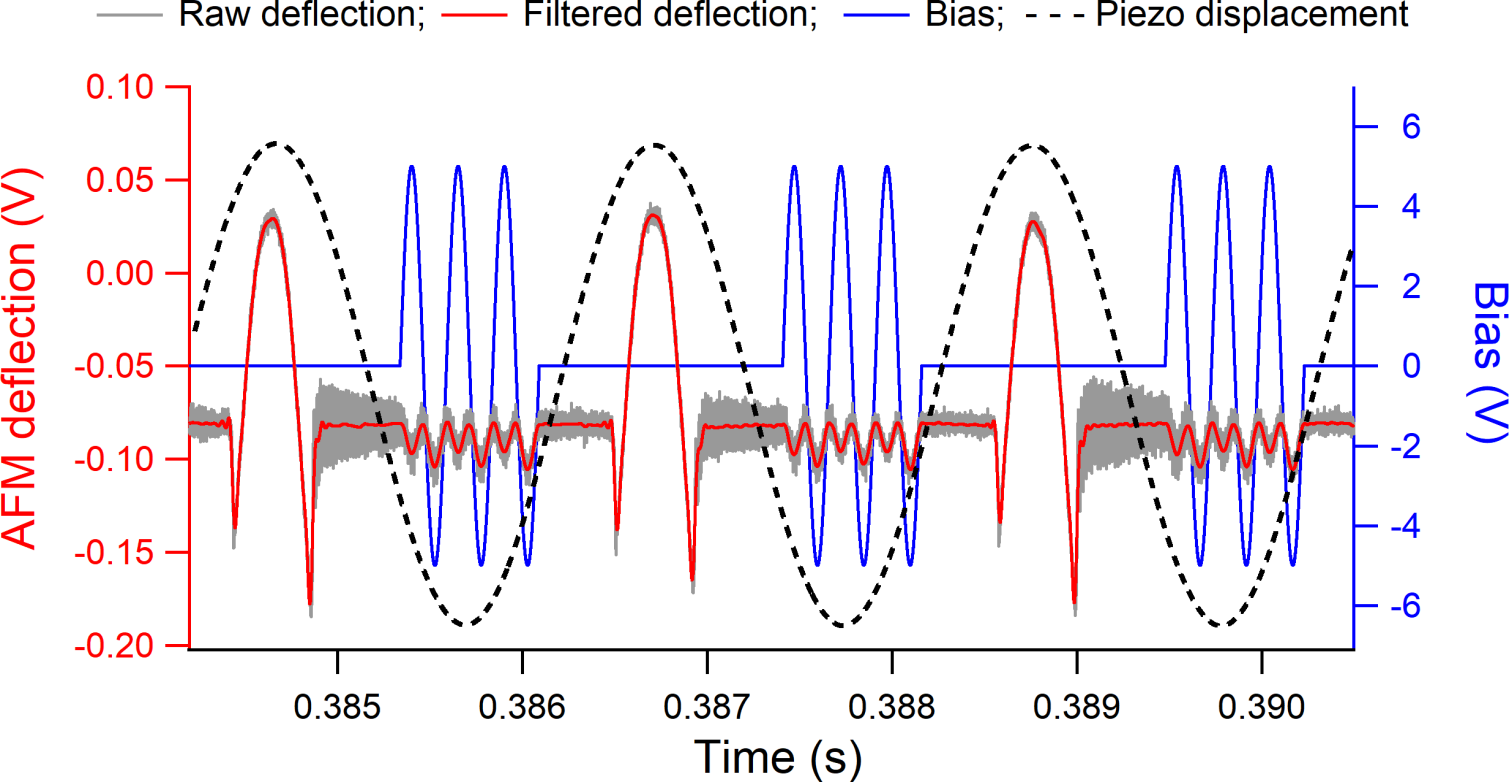
Consecutive taps performed during OP AM-KPFM operated on top of the PFT mode. The bias modulation (blue signal to the right axis) was applied only in between the PFT contacts (raw signal in gray and filtered signal in red to the left axis). The piezo motion (dashed curve) of the PFT was in the form of a sinusoidal oscillation of 50 nm amplitude at 0.5 kHz frequency.

A typical cantilever deflection to the synchronized PFT and KPFM modulations over three consecutive PFT oscillations is shown in Figure 2. The raw PFT signal (gray curve in Figure 2) is especially noisy right after each detachment of the AFM probe from contact due to the relative compliance of the probe used, with a nominal stiffness of 3.0 N/m and first resonance frequency of 67 kHz. This extra high-frequency and small-amplitude ringing was low-pass filtered at 25 kHz, which is a few times larger than the bias frequency, such that the CPD calculations will not be affected by this filter, but below the first resonance frequency to remove the induced ringing. The filtered signal (red curve in Figure 2) highlights the deflection of the cantilever to the bias excitation and shows no perturbation of the PFT motion due to intertwining with the bias modulation. Because the bias voltage is applied only during the out-of-contact intervals, any electrical discharge at the contacts between the conductive tip and sample is prevented. The previously proposed one-pass CL-KPFM implementations operating on top of a PFT-type mode used a continuous KPFM modulation during the out-of-contact intervals of the PFT oscillation [65–66]. This might affect the snap-in and pull-off acts of the PFT motion and, being operated in the proximity of the surface, might inadvertently include contributions from the van der Waals tip–sample interaction to the measured CPD. In the current OL KPFM-PFT implementation, all these impediments are avoided by precisely controlling the synchronization of the bias modulation with the PFT motion in terms of time duration and positioning of the bias signal within the out-of-contact intervals of the PFT cycle. Additionally, the tip–sample separation at which the bias is applied and the CPD is measured is controlled by the amplitude of the PFT oscillation.

The applied bias modulation of the current OL AM-KPFM was synthesized a priori in LabView (National Instruments, Austin, TX, USA) as a sinusoidal waveform with the desired amplitude and frequency that was later synchronized with the PFT oscillation. A typical configuration used in this work was with a bias of 5.0 V amplitude and 4.0 kHz frequency although smaller voltages as low as 2.0 V and higher frequencies up to 30 kHz were found to work as well; no bias frequencies around or above the first resonance frequency of the cantilever were tested. Applied bias, piezo signal, and AFM deflection were simultaneously sampled at 3.0 MSa/s by a high-speed data acquisition board. The PFT scans were performed over 25 μm to encompass large Al and Au regions on each side of the trench, with a PFT modulation of 50 nm amplitude and 0.5 kHz frequency. As can be seen in Figure 2, a characteristic feature of the OL-KPFM measurements is the direct correlation between the magnitude of the tip–sample CPD and the asymmetric response of the cantilever deflection to a sinusoidal bias voltage, namely the change in amplitude with the sign of the bias gradient. This dependence is observed in Figure 2 over an Al region where the measured tip–sample CPD was about 0.9 V; the asymmetry cancels out when the CPD approaches zero.

The AFM probe–sample configuration for KPFM measurement purpose is approximated to that of a plane capacitor, with the electrostatic force between the two electrodes having a parabolic voltage dependence,

[1] $$F=\text{CF}\cdot {\left(V-\text{CPD}\right)}^{2},$$

with *V* the applied bias voltage, CPD the contact potential difference between the AFM probe and sample, and CF the capacitive factor depending on the geometry and dielectric properties of the system. Expressions of CF are obtained from the detailed calculation of the electrostatic force between the AFM probe and sample [67–69]. When the bias is in the form of a sinusoidal modulation, such as *V*_m_·sin(2π*f*_m_*t*) with amplitude *V*_m_ and frequency *f*_m_, the time dependence of the capacitive force can be explicitly observed as

[2] $$F=\text{CF}\cdot {\left[V_{\text{m}}\sin\left(2\pi f_{\text{m}}t\right)-\text{CPD}\right]}^{2}.$$

**Figure 3:**
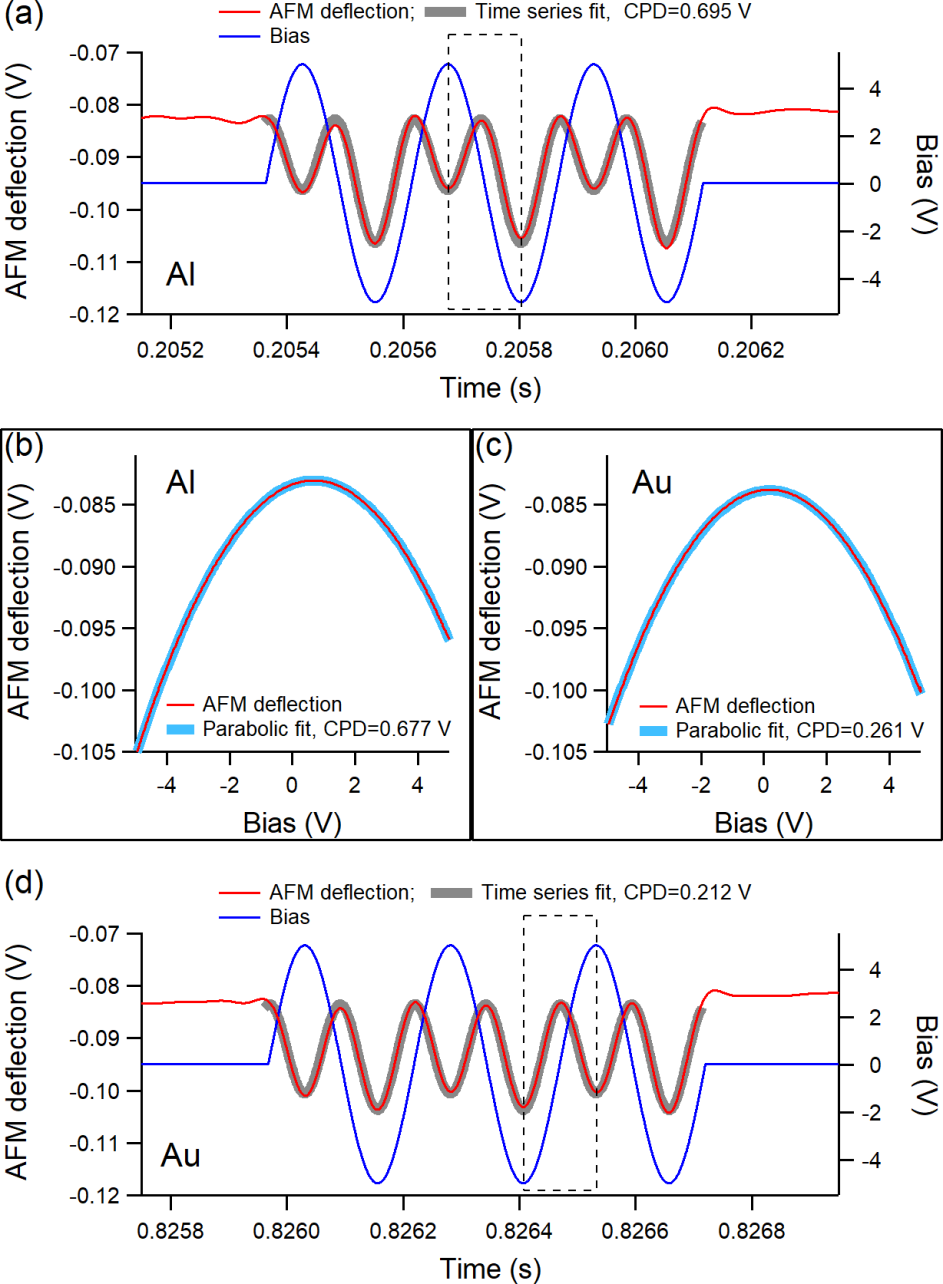
The AFM deflection response to three sinusoidal pulses of bias modulation during the out-of-contact time between two consecutive taps over (a) Al and (d) Au; the thick highlighted gray curves in both (a) and (d) are fits from the times series dependence of the AFM deflection in each case; (b) parabolic fit of the bias dependence of the AFM deflection within the box shown in (a); (c) parabolic fit of the bias dependence of the AFM deflection within the box shown in (d). The measurements were carried out as OL AM-KPFM with the cantilever over the Au region.

Equation 1 and Equation 2 provide two nominally equivalent ways of extracting the CPD from the bias dependence of the AFM deflection and examples of fits using them are shown in Figure 3 at two locations over the Al and Au regions.

To extract the CPD from Equation 1, a parabolic fit is applied on the bias dependence of the AFM deflection as shown in Figure 3b and Figure 3c, with the voltage *V* as variable and CF and CPD as fit parameters. Because the bias oscillation applied at any location in the scan consists of three cycles, five full ramps with the bias going through 0 (either from +*V*_m_ to −*V*_m_ or from −*V*_m_ to +*V*_m_) were selected. For the bias modulation shown in Figure 3a on Al, the CPD determined from the five individual parabolic fits were 0.715, 0.721, 0.677 (shown in Figure 3b), 0.689, and 0.736 V, which give an average value of 0.708 ± 0.024 V. Similarly, the parabolic fits of the bias modulation shown in Figure 3d provided the values of 0.182, 0.266, 0.195, 0.261 (shown in Figure 3c), and 0.229 V, which give an average value of 0.227 ± 0.037 V for the CPD at that location on Au. In general, the scattering of the CPD determined from the five parabolic fits of each bias train, either on Al or Au, was within 0.050 V; here and in the rest of the text, the uncertainties represent one standard deviation from the average value.

The fit for the time series dependence of the AFM deflection under the sinusoidal bias modulation (refer to Equation 2) uses two fixed parameters (the amplitude *V*_m_ and the frequency *f*_m_), two fit parameters (CF and CPD), and the time *t* as variable. For the examples shown in Figure 3, the time series fits (gray curves) are superimposed over the actual data and the CPD values determined from them were 0.695 ± 0.03 V and 0.212 ± 0.002 V for Al and Au, respectively. These results point to a much smaller uncertainty for the determined CPD from time series analysis in comparison to that from the parabolic fits [36]. The time series analysis applies on the entire train of pulses and provides a more global characterization of the KPFM response at each location.

**Figure 4:**
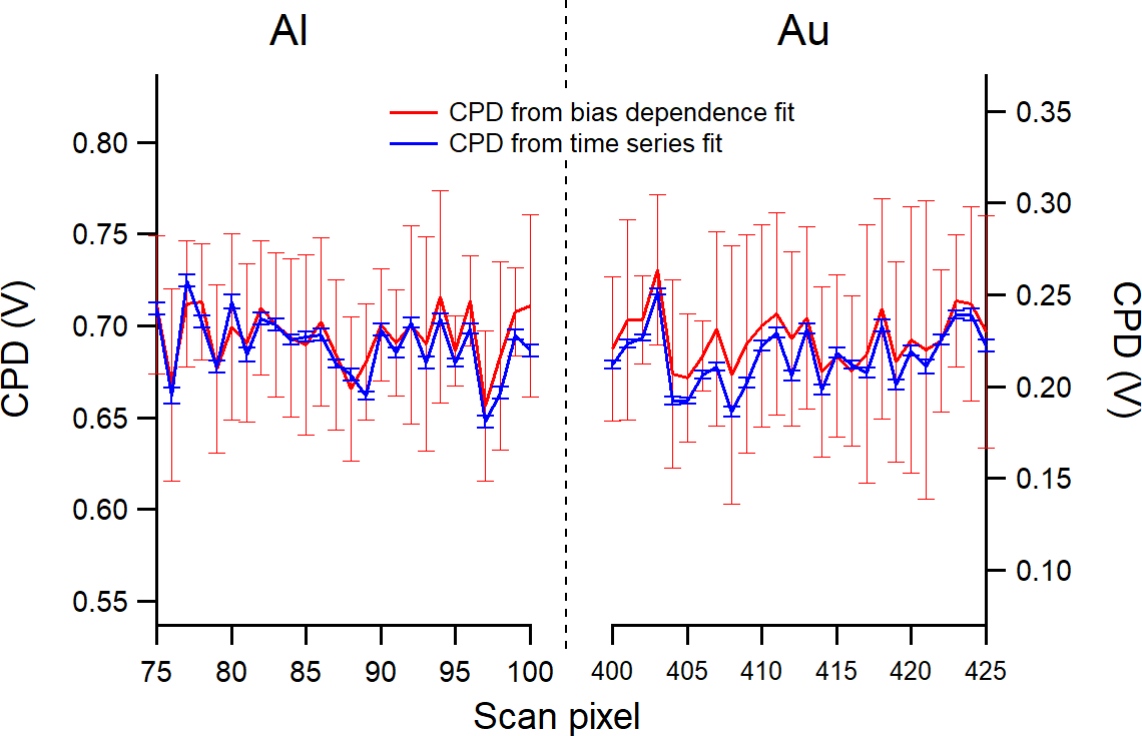
Average and standard deviation values of CPD determined from parabolic bias dependence and time series dependence of the AFM deflection during a scanning line over some Al and Au regions.

The comparison of the two analyses along parts of a scan line across Al and Au is shown in Figure 4. As can be seen, both the parabolic and time series fits provide similar average values of the CPD on Al (left) and Au (right). However, the uncertainties are much smaller with the times series analysis than those of the parabolic analysis. In the following, the time series results will be used primarily for measurement interpretation and mapping reconstruction.

### Assessment of the local CPD from OL AM-KPFM data

Benefiting from the data availability of the OL AM-KPFM, appropriate models for the electrostatic interaction between the AFM probe and sample can be introduced in the data analysis. A simple consideration for the non-local contribution of the cantilever–sample coupling to the measured CPD is to assume the AFM probe (tip + cantilever) as two capacitors in series and add the two parabolic voltage dependences to the total electrostatic force:

[3] $$F=\text{TCF}\cdot {\left(V-{\text{CPD}}_{\text{local}}\right)}^{2}+\text{CCF}{\left(V-{\text{CPD}}_{\text{non-local}}\right)}^{2}.$$

In this formulation, the AFM tip “sees” a local coupling characterized by CPD_local_ whereas the cantilever contributes with an average background component, CPD_non-local_; TCF and CCF are the distinct tip capacitive force and cantilever capacitive force coefficients, respectively. Similarly, in the time series analysis, the total electrostatic force is described by

[4] $$\begin{array}{c} F=\text{TCF}\cdot {\left[V_{\text{m}}\sin\left(2\pi f_{\text{m}}t\right)-{\text{CPD}}_{\text{local}}\right]}^{2}\\ \text{+ CCF}\cdot {\left[V_{\text{m}}\sin\left(2\pi f_{\text{m}}t\right)-{\text{CPD}}_{\text{non-local}}\right]}^{2}, \end{array}$$

with the same parameters as in Equation 3.

For a reliable deconvolution of the cantilever contribution by either Equation 3 or Equation 4, fixed values or constraints must be applied on the two additional fit parameters CCF and CPD_non-local_. These can come either from calculation or additional measurements. In the present case, CPD_non-local_ is considered constant and equal to the average CPD of the region over which the cantilever is located, namely 0.21 V when the cantilever is over the Au region and 0.93 V when the cantilever is over the Al region. These were the average CPD values obtained from preliminary OL AM-KPFM when the entire AFM probe (cantilever and tip) was on each of these regions, in which case CPD_local_ = CPD_non-local_. It is worth pointing out that these values match the ones from CL AM-KPFM and CL FM-KPFM when the scans were made with the entire AFM probe over each of the Au and Al regions. Once the value of CPD_non-local_ is ascribed, the CCF can be obtained by rescaling the OL AM-KPFM traces to match a known value of the CPD_local_. The strip geometry of the investigated sample offers a good test vehicle for such rescaling and it can be used in general for preliminary calibrations. The value determined for CPD_non-local_ becomes less accurate when the region underneath the cantilever has large variations in surface potential. By adjusting the relative orientation of the cantilever with respect to the scanned area (i.e., sample rotation or scanning at a different angle), an average value of CPD_non-local_ can still be negotiated for the region where the cantilever mostly resides during the actual scanning. Conceivably, there might be some cases (e.g., radial patterns with pitch smaller than the length of the cantilever) when the proposed analysis would be less effective.

**Figure 5:**
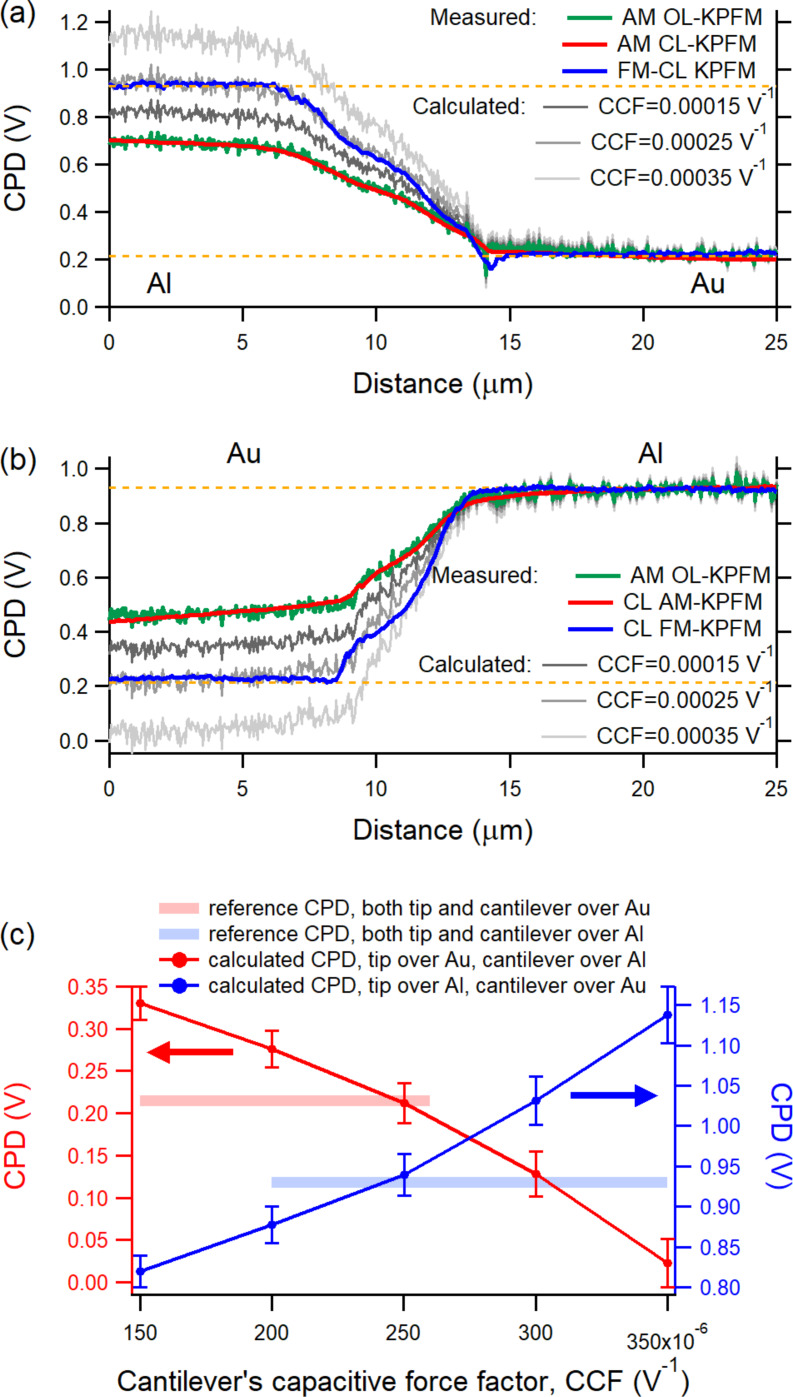
Comparison of CPD traces from OP AM-KPFM, CL AM-KPFM, and CL FM-KPFM in both measurement configurations, with the cantilever over (a) the Al region and (b) the Au region. The CPD determined from OL AM-KPFM measurements is rescaled differently by the fit parameter CCF that amounts for the average contribution of the cantilever (see text for details); (c) the same CCF = 0.000250 V^−1^ is found to adjust the average CPD values over Au and Al to their reference values of 0.21 V and 0.93 V, respectively.

Figure 5a (cantilever over Al) and Figure 5b (cantilever over Au) show the measured and rescaled CPD traces of the OL AM-KPFM along with their counterparts from CL AM-KPFM and CL FM-KPFM over the Au and Al regions with the Si trench between them. In both cases, the CPD determined from OL AM-KPFM by a single time series fit (Equation 2) matches that from CL AM-KPFM over each distinct material region. With CPD_non-local_ fixed in Equation 4 (0.93 V in Figure 5a and 0.21 V in Figure 5b), different CPD traces were calculated for different values of CCF. As can be seen in both Figure 5a and Figure 5b, the calculated CPDlocal values change over the regions where the cantilever is not located (Au in Figure 5a and Al in Figure 5b) but remain unchanged over the region where the cantilever resides (Al in Figure 5a and Au in Figure 5b). To univocally determine the value of CCF, concurrent plots of the rescaled CPD_local_ on both Au and Al regions where observed as a function of CCF. As can be seen in Figure 5c, the calculated CPD_local_ of both Au and Al can be adjusted to their reference values, 0.21 V for Au and 0.93 V for Al, for the same value of CCF, which was 0.000250 V^−1^. The CPD traces from CL AM-KPFM and CL FM-KPFM across the Au/Si/Al trench are used in Figure 5a and Figure 5b for visual guidance only. The same reference values, 0.21 V for Au and 0.93 V for Al, necessary for rescaling, were obtained from preliminary OL AM-KPFM scans over each region.

**Figure 6:**
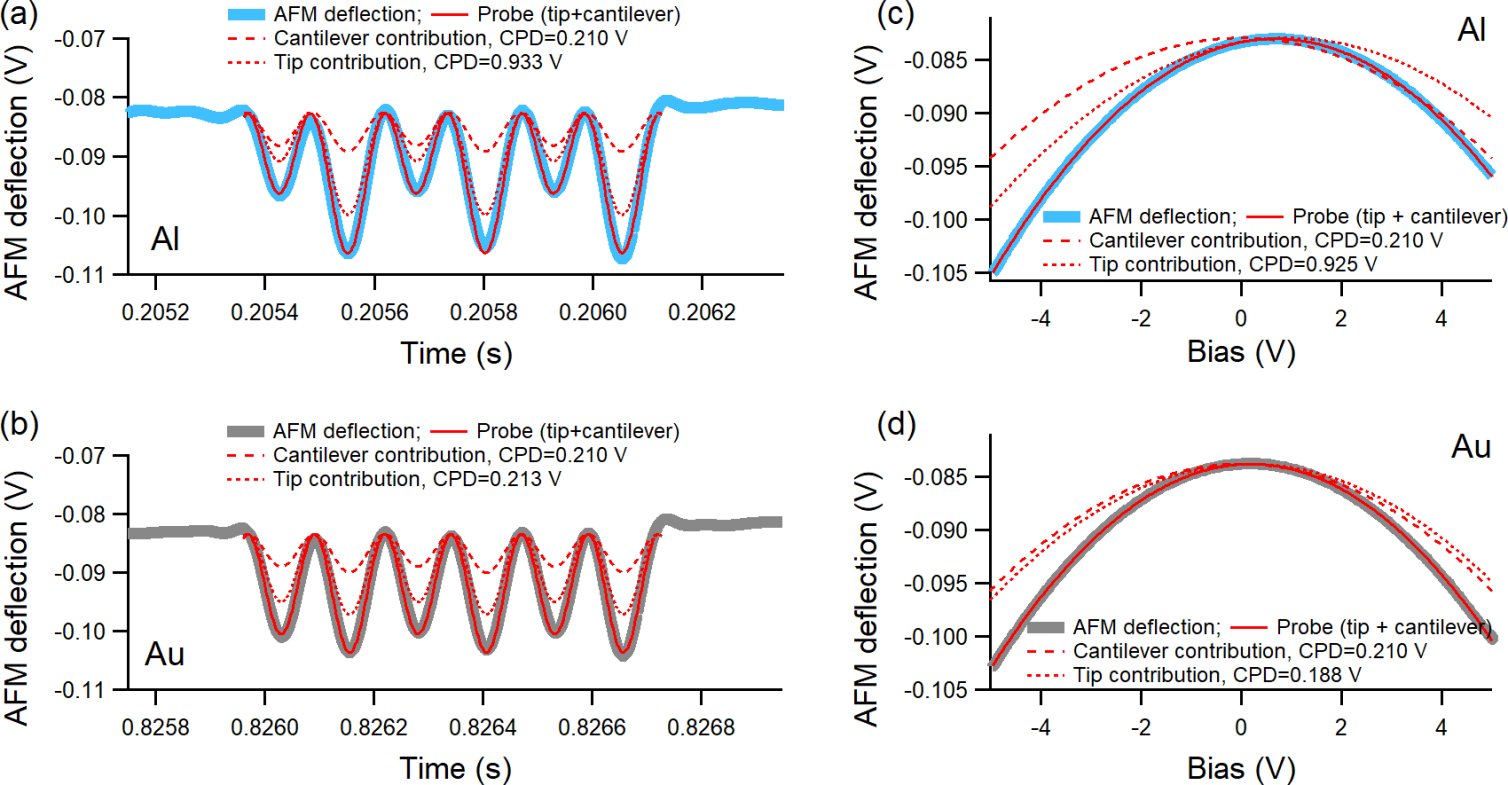
Separation of the average contributions of the tip and cantilever to the measured OL AM-KPFM signal: (a, b) time series fits of the bias-modulated AFM deflections shown in Figure 3a and Figure 3d, respectively; (c, d) parabolic fits of the bias-dependent AFM deflections shown in Figure 3b and Figure 3c, respectively.

With the two fit parameters CCF and CPD_non-local_ fixed from rescaling OL AM-KPFM measurements on known references, CPD_local_ can be deconvoluted from the measurements by using one of Equation 3 or Equation 4 and assembled into maps. Figure 6 shows the tip and cantilever contributions to the fits of the same traces that were analyzed in Figure 3 by single time series and bias parabolic contributions. In Figure 6a and Figure 6b, the time series fits are shown on Al and Au, respectively. As can be seen, a large contribution to the modulated AFM deflection comes from the cantilever. This is due to the long-distance nature of the electrostatic force, which makes the cantilever–sample interaction significant even if the cantilever is about 15 μm above the sample; the cantilever–sample distance is mainly imposed by the height of the tip. In Figure 6c and Figure 6d, the parabolic contributions of the tip and cantilever are shown on deflection-versus-bias plots on both Al and Au (same AFM deflections as in Figure 3b and Figure 3c). In this representation, the distinct values of CPD_local_ and CPD_non-local_ are observed in shifted parabolas with different apex locations.

**Figure 7:**
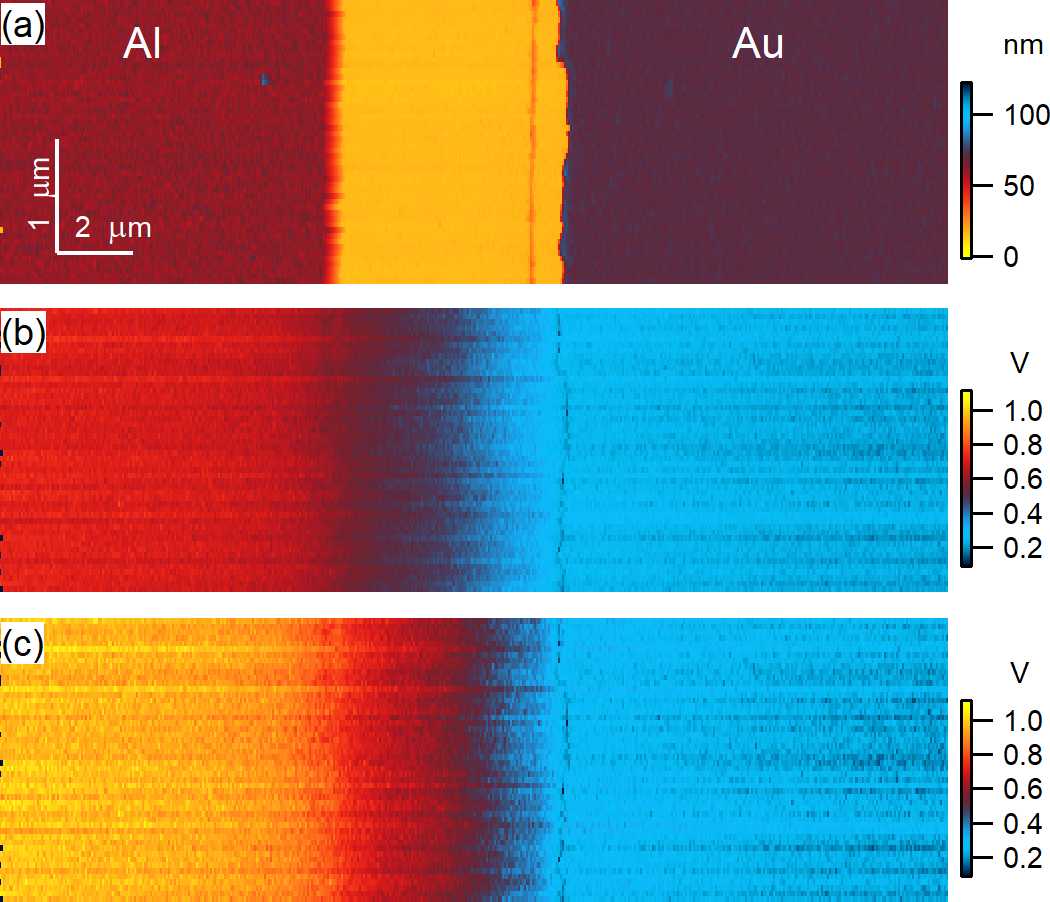
Maps from OL AM-KPFM operated in PFT mode over an Al/Si/Au trench: (a) topography extracted from the measured piezo signal of the PFT; (b) OL AM-KPFM map over (a) with the cantilever contribution included; (c) OL AM-KPFM map over (a) with cantilever contribution subtracted.

The CPD maps from both convoluted and deconvoluted OL AM-KPFM measurements are shown in Figure 7 over a scanned area encompassing Al and Au regions on each side of a Si trench; in this case the cantilever was located over the Au part. The topography (Figure 7a) was retrieved from the piezo displacement signal included in the bundle of the OL AM-KPFM measurements. The benefit of measuring both the electrical (KPFM) and mechanical (PFT) signals provides a pixel-by-pixel correlation between CPD and topography. Prior to subtracting the cantilever contribution, the average CPD over Al from OL AM-KPFM (left side of Figure 7b) was 0.7 V, the same as that from CL AM-KPFM mapping in the same configuration (cantilever over the Au region). After deconvolution, the average CPD of the Al region was brought to about 0.9 V, the same as in the CL FM-KPFM maps. The deconvolution does not affect the CPD value of the region over which the cantilever is during scanning, in this case the Au region.

**Figure 8:**
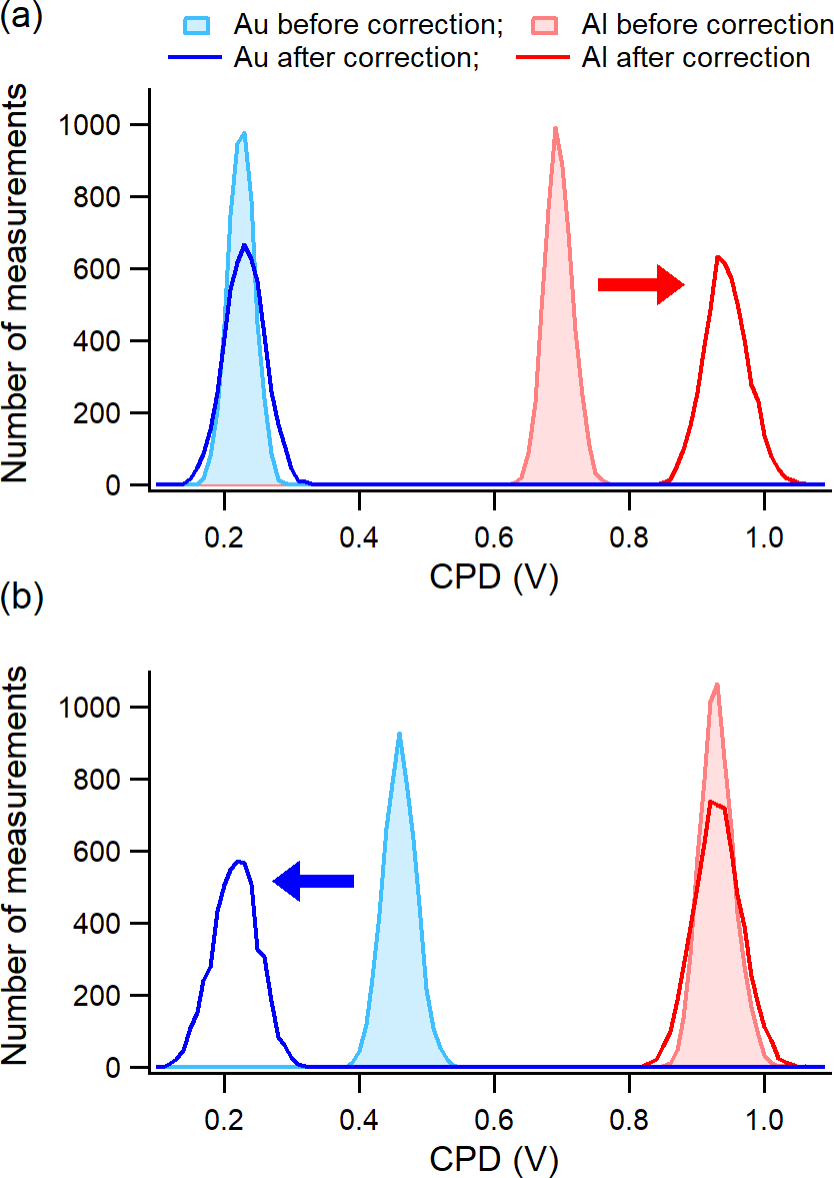
Histograms of the OL AM-KPFM measurements over the Al and Au regions: (a) The measurements were made with the cantilever over the Au region. The CPD over the Al region was corrected after the cantilever contribution was subtracted (data were collected from the maps shown in Figure 7b,c); (b) The measurements were made with cantilever over the Al region. The CPD over the Au region was corrected after the cantilever contribution was subtracted. The arrows indicate the changes made by these corrections to the determined CPD values.

The results of the OL AM-KPFM maps from both configuration measurements are summarized in Figure 8 in the form of histograms. The histograms of the maps discussed in Figure 7 are shown in Figure 8a. In Figure 8b, the measurements from the configuration with the cantilever over Al are shown before and after deconvolution. In this case, the local CPD between tip and sample was rescaled over Au from 0.5 to 0.2 V. For the two considered cantilever positions, the rescaled CPD values from OL AM-KPFM on both Au and Al regions match those shown in Figure 1h from CL FM-KPFM for either of the cantilever positions and from CL AM-KPFM when the cantilever was over the same region as the tip.

## Conclusion

In this work, a new OL AM-KPFM method was demonstrated in the single-pass scanning of the PFT mode. A sinusoidal bias modulation was synchronized with the out-of-contact intervals of the PFT motion to perform OL AM-KPFM measurements at each location in the scan. The high-speed digitization of the piezo displacement, AFM deflection, and applied bias signals provided detailed observation of the tip–sample mechanical and electrical interactions for topography reconstruction and KPFM characterization. The AFM response to the bias modulation was analyzed both by parabolic and time series dependencies. With each of these analyses the cantilever contribution to the determined CPD was evaluated based on known references. This brought the accuracy of the AM-KPFM measurement over a heterostructure to the same level as that of its FM-KPFM counterpart. The advantage of using OL AM-KPFM resides in the direct and easy implementation of the measurements with the possibility of accommodating relevant descriptions of the electrostatic interactions probed over various regions of a sample.

## Disclaimer

Certain commercial equipment, instruments, or materials are identified in this document. Such identification does not imply recommendation or endorsement by the National Institute of Standards and Technology, nor does it imply that the products identified are necessarily the best available for the purpose.
